# Associations of Heat-Shock Protein Expression with Meat Quality and Sensory Quality Characteristics in Highly Marbled *Longissimus Thoracis* Muscle from Hanwoo Steers Categorized by Warner–Bratzler Shear Force Value

**DOI:** 10.3390/foods8120638

**Published:** 2019-12-04

**Authors:** Eunmi Oh, Boin Lee, Young Min Choi

**Affiliations:** Department of Animal Science, Kyungpook National University, 2559 Gyeongsang-daero, Sangju-si, Gyeongsangbuk-do 37224, Korea; eunmi95@knu.ac.kr (E.O.); ananassab@knu.ac.kr (B.L.)

**Keywords:** heat-shock protein, eating quality, tenderness, highly marbled beef, Hanwoo steers

## Abstract

The influence of heat-shock protein (HSP) concentrations at 45 min and 24 h postmortem on meat quality and sensory quality characteristics of *longissimus thoracis* muscle from highly marbled Hanwoo steers (beef marbling standard grade 6–8) was investigated. Muscle samples were segregated into three groups based on Warner–Bratzler shear force (WBSF) value. The low group exhibited a lower WBSF value compared to the medium and high groups (37.8 vs. 48.9 and 64.3 N, *p* < 0.001). Muscle pH at 45 min and 24 h postmortem was not different (*p* > 0.05), and all groups exhibited low ultimate pH value (pH < 5.8). Beef steaks from the low group were significantly easier to pierce and chew, and they left less perceptible residue than the high group (*p* < 0.05). These differences in tenderness attributes were associated with differences in small HSPs at 45 min postmortem, with the low group exhibiting a lower level of αβ-crystallin and higher levels of HSP20 and HSP27 compared with the high group (*p* < 0.05). No differences were observed for small HSPs, HSP70, and HSP90 at 24 h postmortem (*p* > 0.05). Therefore, the expression levels of small HSPs at 45 min postmortem seems to have the potential to be an indicator of tenderness in highly marbled Hanwoo beef with low ultimate pH.

## 1. Introduction

Heat-shock proteins (HSPs) are well known as molecular chaperones that assist in the normal folding of various polypeptides, help misfolded polypeptides regain their proper configuration, and regulate proteolysis [1,2]. These chaperone properties of HSPs are expressed under a variety of conditions including normal, stressed, and apoptotic, but HSPs do not remain as part of the final protein structure [2]. HSPs are categorized by their functions and molecular weights into distinct families (small HSPs, HSP70, HSP90, etc.), and individual HSPs exhibit different biological functions under apoptotic conditions [2,3]. After exsanguination, muscle fibers convert to an ischemic-anoxic state, and apoptotic cell death occurs before rigor mortis during the conversion of muscle to meat [4]. To maintain cell homeostasis against apoptosis, pro- and anti-apoptotic proteins are released from mitochondria [4]. The levels of HSPs at the early postmortem period are associated with the rate and extent of apoptosis, and they can influence structural and metabolic changes of skeletal muscle during the postmortem period [4,5]. 

For these reasons, variation in HSP levels associated with apoptosis during the postmortem period can affect the meat quality and sensory quality characteristics, especially the extent of meat tenderization [5,6]. The protective chaperoning properties of small HSPs, including αβ-crystallin, HSP20, and HSP27, during apoptotic cell death could delay the rate of protein degradation and beef aging [5]. Bernard et al. [7] and Kim et al. [6] reported a negative correlation between the levels of small HSPs and palatability of cooked beef, especially tenderness. Additionally, numerous studies reported inconsistent tenderness of intermediate pH beef (ultimate pH ranged between 5.8 and 6.2), and they also suggested that the observed toughness of intermediate pH beef is associated with higher concentrations of small HSPs [5,8,9]. Yu et al. [10] reported that poor water-holding capacity (WHC) and faster pH decline in the porcine *longissimus dorsi* muscle were associated with decreased expression levels of HSP70 and HSP90, as these HSPs play important roles in maintaining the structural network of muscle fiber [11].

Hanwoo steers are known to have a higher intramuscular fat (IMF) content, and the beef tends to exhibit higher sensory quality characteristics, especially tenderness, compared with other breeds such as Holstein steers [12,13,14,15]. Inconsistent sensory tenderness is occasionally observed in highly marbled beef, although consumers have high expectations for organoleptic characteristics [14]. The biochemical mechanisms, especially the functions of HSPs during apoptosis, that cause tenderness variation in highly marbled beef are largely unknown. Therefore, the objective of this study was to investigate the relationship between HSP expressions, including αβ-crystallin, HSP20, HSP27, HSP70, and HSP90, and meat quality and sensory quality characteristics of highly marbled Hanwoo steers to identify factors that are associated with tenderness variation in highly marbled beef.

## 2. Materials and Methods

### 2.1. Animals and Muscle Sample Preparation

Hanwoo steers (aged 28–33 months, carcass weight of 466.7 ± 65.6 kg) were transported and slaughtered at the same commercial slaughterhouse in Hoengseong (Korea), and they were randomly selected from a total of 364 highly marbled Hanwoo steers and obtained in three batches (16, 16, and 17 animals per day; total 49 steers). At 45 min postmortem, muscle samples (approximately 20 g per sample) were taken from the *longissimus thoracis* (LT) muscle at the 13th thoracic vertebra (the standard site of carcass grading in Korea), frozen in liquid nitrogen, and stored at −80 °C for Western blot analysis, and muscle pH was measured directly at the standard site of carcass grading. 

At 24 h postmortem, the carcasses were graded according to the quality grading standard, including marbling, of the Korea Institute of Animal Products Quality Evaluation (KAPE) [16]. Each loin was scored on a nine-point scale (1–9, devoid to very abundant) according to the beef marbling standard (BMS), and highly marbled loin cuts (BMS 6–8) were randomly selected and used for the meat quality and sensory quality measurements. The KAPE provided carcass weight, loin eye area, back-fat thickness, and BMS. After quality grading, samples were collected between the ninth and 13th thoracic vertebrae of the LT muscle, and meat quality measurements including Warner–Bratzler shear force (WBSF) were immediately performed. For analysis of eating quality, each muscle sample was cut into steak-sized chops (1.5 cm thickness, approximately 100 to 120 g), and then immediately frozen and stored at −20 °C. Additionally, approximately 50 g samples were taken and stored at −80 °C before measuring the IMF concentration and performing Western blot analysis. The IMF content was determined by the Soxhlet method [17] using a solvent extraction system.

### 2.2. Warner–Bratzler Shear Force

WBSF analysis was performed based on a previously published method [18]. Beef samples of approximately 80 g were individually placed inside a polyethylene bags and heated in a water bath (80 °C) until the core temperature reached 71 °C as measured by a spear-type thermometer (Testo 108, Testo Inc., Lenzkirch, Germany). For analysis of WBSF, more than 10 replicate cores (1.27 cm in diameter) were analyzed by an Instron Universal Testing Machine (Model 1011, Instron Corp., Canton, OH, USA) equipped with a Warner–Bratzler blade operating at a crosshead speed of 200 mm/min [19].

### 2.3. Meat Quality Characteristics

The muscle pH_45 min_ and pH_24 h_ were determined using a portable pH instrument with a penetration probe (Testo 206-pH2, Testo Inc., Lenzkirch, Germany). Meat surface color was assessed using a Minolta chromometer (CR-400, Minolta Camera Co., Osaka, Japan), which uses diffuse D65 standard illumination and a 0° viewing angle, on beef loin surfaces after blooming at 4 °C for 30 min. The lightness (*L* ^*^), redness (*a* ^*^), and yellowness (*b* ^*^) values were expressed as the recommendations of Commission Internationale de l’Eclairage (C.I.E.) [20]. To measure drip loss, muscle samples of approximately 80 g were put into plastic bags and hung on a net, ensuring that the samples were not in contact with the plastic bag for 48 h at 4 °C. Percentage of drip loss was calculated as the sample weight change [18]. For measurement of filter-paper fluid uptake (FFU), the filter paper was pre-weighed and placed on the meat surface for less than 2 s to absorb fluids and then weighed [21]. The cooking loss was also calculated by weighing before and after cooking [18]. Preparation of cooked meat samples for both the WBSF and cooking loss analysis was the same.

### 2.4. Sensory Quality Evaluation

For sensory quality evaluations, a total of 147 beef samples (49 muscle samples × 3 replicates) were used during 30 sessions (4–5 loin steaks per session). Eleven trained panelists (six females and five males; 24 to 40 years of age) were used in this study. Approval was granted by the Bioethics Committee of Kyungpook National University (protocol number 2019-0027). Panelist training and sensory quality evaluations were conducted at the Muscle Biology Laboratory of Kyungpook National University under the guidelines of the American Meat Science Association [19] and Meilgaard et al. [22]. The steak samples were thawed at 4 °C for 24 h and roasted in a convection oven (MJ324, LG Electronics, Seoul, Korea), which was preheated to 180 °C. The samples were turned every 3 min and cooked until their core temperatures reached 71 °C, as measured by a thermometer (Testo 108, Testo Inc., Lenzkirch, Germany). Cooked beef was trimmed of surface meat and cut into 1.3 cm^3^ cubes. Panelists evaluated nine attributes and were instructed to refresh their palate between each tasting with unsalted crackers and water. Scores of tenderness attributes, mouth coating, juiciness, flavor, and off-flavor intensities ranged from 1–9 (low to high) based on previously published procedures [13,19,22].

### 2.5. Immunoblotting

Sarcoplasmic protein was extracted at 45 min and 24 h postmortem, according to Pietrzak et al. [23] with slight modifications. The protein concentration was measured by a Bio-Rad protein assay kit (Bio-Rad Laboratories Inc., Richmond, CA, USA) according to the Bradford method [24]. Sarcoplasmic proteins were separated by SDS-PAGE using a Mini-PROTEAN system (Bio-Rad Laboratory Inc., Richmond, CA, USA), transferred to polyvinylidene fluoride (PVDF) membranes (GE Healthcare Ltd., Freiburg, Germany), and blocked with 5% non-fat dry milk powder in Tris-buffered saline (TBS)/Tween. For Western blot analysis, the primary antibodies used were αβ-crystallin (1:10,000 dilution; ab13496, Abcam Ltd., Cambridge, UK), HSP20 (1:400 dilution; sc-51955, Santa Cruz Biotechnology Inc., Santa Cruz, CA, USA), HSP27 (1:3000 dilution; sc-13132, Santa Cruz Biotechnology Inc., Santa Cruz, CA, USA), HSP70 (1:1000 dilution; SPA-820, StressGen Biotechnology Corp., Victoria, Canada), and HSP90 (1:2000 dilution; SPA-835, StressGen Biotechnology Corp., Victoria, Canada). Secondary antibodies were anti-mouse immunoglobulin G (IgG) horseradish peroxidase (HRP)-linked antibody (1:3000 dilution for αβ-crystallin, HSP20, HSP27, and HSP70; Cell Signaling Technology, Inc., Beverly, MA, USA) and anti-mouse m-IgGκ binding protein (BP)/HRP (1:3000 dilution for HSP90; Santa Cruz Biotechnology Inc., Santa Cruz, CA, USA). Proteins were detected with a WesternBright ECL kit (Advansta Inc., Menlo Park, CA, USA) and imaged using an ImageQuant LAS 500 (GE Healthcare Ltd., Freiburg, Germany). Image analysis for each protein band was performed using one-dimensional (1D) image analysis software (Eastman Kodak Co., Rochester, NY, USA). The intensities of protein bands in the same gel were compared, and they were normalized by band intensities of the low group.

### 2.6. Statistical Analysis

Cluster analysis was conducted using the FASTCLUS procedure of SAS software 9.4 (SAS Institute Inc., Cary, NC, USA) to generate three clusters (low, *n* = 17; medium, *n* = 18; high, *n* = 14) based on WBSF value. Normality check was performed by SAS PROC UNIVARIATE. Regarding the WBSF value, BMS, IMF content, carcass traits, meat quality characteristics, and expression levels of HSPs, the general linear model (GLM) procedure was used to elucidate any associations. Regarding the sensory quality characteristics, a GLM was performed to analyze the WBSF value as a fixed effect, while the trained panelists were considered as random effects. Significant differences in the least-square means (LSMs) of investigated parameters between groups were compared by the probability difference option at *p* ≤ 0.05. All data were presented as LSMs together with the standard errors.

## 3. Results

### 3.1. Comparison of WBSF, BMS, IMF Content, and Carcass Characteristics between WBSF Groups

Table 1 presents the comparison of WBSF value, BMS, IMF content, and carcass characteristics between groups categorized by WBSF value. As expected, a marked difference was observed in WBSF value of the LT muscle between the groups (*p* < 0.001), and the high group exhibited the highest WBSF value compared with the medium and low groups (64.3 vs. 48.9 and 37.8 N, respectively). There were no significant differences in BMS assessed by trained graders and IMF concentration between the groups (*p* > 0.05). Regarding the carcass characteristics, no differences were observed for carcass weight and loin eye area between the groups, although the high group showed a thinner back-fat value at the 13th thoracic vertebra compared with the low and medium groups (13.8 vs. 18.8 and 19.5 mm, respectively, *p* < 0.05).

### 3.2. Comparison of Meat and Sensory Quality Characteristics between WBSF Groups

Beef quality characteristics of the Hanwoo LT muscle for WBSF groups are presented in Table 2. There were no significant differences in muscle pH_45 min_ and pH_24 h_ between WBSF groups (*p* > 0.05). Measured meat color, including lightness, redness, and yellowness, also did not differ between the groups (*p* > 0.05). The high group did exhibit a higher cooking loss compared with the low group (21.7 vs. 17.1%, *p* < 0.05), although no differences between the groups were observed in other WHC measurements (*p* > 0.05).

There were significant differences in all tenderness attributes between WBSF groups (Table 3). A noticeable difference was detected in sensory softness between the groups, and beef samples with a higher WBSF value exhibited a lower softness score compared to beef samples with a lower WBSF value (6.16 vs. 7.49, *p* < 0.001). Samples from the high group received lower scores for initial tenderness, chewiness, rate of breakdown, and amount of perceptible residue compared with samples from the medium group (*p* < 0.05). Higher scores of mouth coating (6.97 vs. 6.26, *p* < 0.01) and juiciness (6.69 vs. 5.85, *p* < 0.01) were observed in the low group compared with the high group. However, no significant differences were observed in flavor and off-flavor intensities between WBSF groups (*p* > 0.05).

### 3.3. Comparison of HSP Expression Levels between WBSF Groups

**Figure 1:**
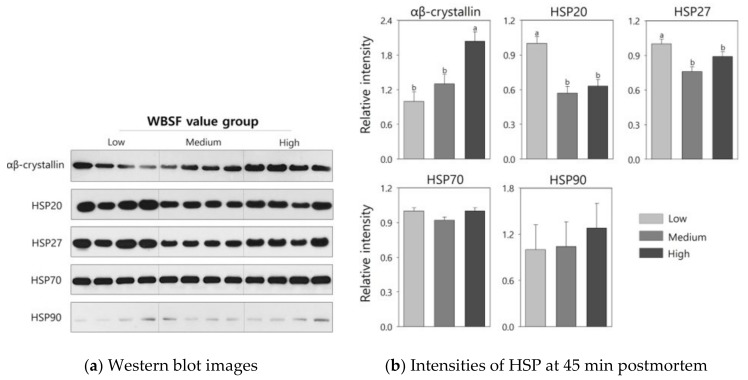
Western blot images (**a**) and relative intensities (**b**) of the indicated heat-shock proteins (HSPs) in the Hanwoo *longissimus thoracis* muscle at 45 min postmortem for groups categorized by Warner–Bratzler shear force (WBSF) value. Bars indicate standard errors of the means. ^a–b^ Different letters denote significant differences (*p* < 0.05).

**Figure 2:**
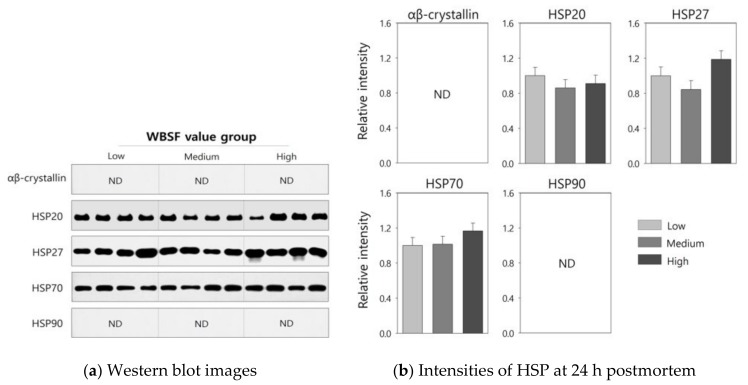
Western blot images (**a**) and relative intensities (**b**) of the indicated heat-shock proteins (HSPs) in the Hanwoo *longissimus thoracis* muscle at 24 h postmortem for groups categorized by Warner–Bratzler shear force (WBSF) value. Bars indicate standard errors of the means. ND, not detected.

HSP levels in the LT muscle at the early postmortem period were assessed by Western blot analysis, and the results are graphically shown in Figure 1. The ab13496 antibody specifically reacted with αβ-crystallin in the LT muscle from Hanwoo steers, and expression levels were significantly higher in the high group compared with the low and medium groups (2.04 vs. 1.00 and 1.30, *p* < 0.05). On the other hand, the high group exhibited lower levels of HSP20 (0.62 vs. 1.00, *p* < 0.05) and HSP27 (0.89 vs. 1.00, *p* < 0.05) compared to the low group. However, there were no significant differences in the expression levels of HSP70 and HSP90 between WBSF groups (*p* > 0.05). At 24 h postmortem, αβ-crystallin and HSP90 were not detected in the LT muscle from the Hanwoo steers (Figure 2). There were no significant differences in the expression levels of HSP20, HSP27, and HSP70 at 24 h postmortem between WBSF groups (*p* > 0.05).

## 4. Discussion

Beef palatability, including tenderness, juiciness, and flavor, is one of the most important factors that influence the purchase and repurchase decisions of consumers [25]. In the meat industry, it is important to produce beef with superior eating quality characteristics; it is also important to produce consistently tender beef [25]. Generally, the IMF content or marbling degree is closely associated with the sensory quality characteristics of cooked beef; thus, these characteristics are integral to the beef quality grading systems in the United States, Japan, and Korea [26]. Hanwoo cattle with a higher BMS exhibited more tender, juicy, and flavorful beef compared with cattle with a lower BMS [14,27], and marked differences were also observed in sensory tenderness and WBSF value among the beef quality grades that are primarily based on the BMS [14]. In the current study, BMS and IMF content were similar between the groups categorized by WBSF value, as highly marbled beef loins (BMS 6–8; beef quality grade 1^+^ to 1^++^) were selected. However, significant variation was observed in WBSF value, and beef samples from the low group required less force to pierce the meat sample compared with beef samples from the high group.

Ultimate muscle pH (pH_u_) is associated with inconsistent tenderness [2,5,9]. Beef with intermediate pH_u_ tends to show a higher shear force value compared to beef with high and low pH_u_, as less extensive degradation of cytoskeletal proteins and delayed tenderization are observed in beef with intermediate pH_u_ [2,5,9]. Such intermediate pH_u_ beef was frequently observed in bulls due to low-calorie diets and stress before slaughter [9]. However, highly marbled cattle can be obtained through a longer fattening period with a high-protein diet; therefore, the muscle pH_u_ of Hanwoo steers was generally lower compared with muscle from cattle that were raised in a pasture-fed production system [14]. In the present study, no muscle samples had intermediate pH_24 h_ values; all samples at 24 h postmortem had low pH_24 h_ values (pH < 5.8) (data not shown). Muscle pH values at 45 min and 24 h postmortem were not different between WBSF groups. However, beef with a lower WBSF value exhibited a lower cooking loss compared to beef with a higher WBSF value, as WBSF value was well correlated with cooking loss [18].

There are three aspects to the overall sensation of tenderness: (1) the initial force of teeth penetrating the meat, (2) the ease of fragmentation during chewing, and (3) the amount of perceptible residue after chewing [28,29]. Among the sensory tenderness attributes in this study, the first perception of tenderness was related to softness and initial tenderness, the second aspect was associated with chewiness and rate of breakdown, and the third aspect was associated with the amount of perceptible residue [19,28]. Thus, the WBSF value as an objective tenderness measurement was more closely associated with the first aspect, especially softness, than the second and third aspects. In this study, there was a significant difference in sensory softness between WBSF groups, although no differences were observed in the other tenderness attributes between the low and medium groups. Moreover, there were no differences in mouth coating and flavor intensity between the medium and high groups.

The first phase in the conversion of muscle into meat is the onset of apoptosis, and the biochemical and structural changes that occur during apoptotic cell death after slaughter influence the beef tenderization processes [2,4]. During apoptotic cell death, αβ-crystallin plays an important role in inhibiting the proteolytic activity of caspase 3, a cysteine peptidase associated with apoptosis [30]. Due to the chaperoning property of αβ-crystallin, an increase in the level of this protein leads to reduced proteolytic degradation of myofibrillar proteins, which means the meat is less tender [7]. Lomiwes et al. [31] reported less degradation of cytoskeletal proteins, including titin and desmin, after adding αβ-crystallin to myofibrillar extracts. These results support the data on αβ-crystallin levels in this study, and beef with lower WBSF values exhibited lower level of αβ-crystallin at 45 min postmortem compared with beef with higher WBSF values. However, significantly higher levels of HSP20 and HSP27 were observed in the low group compared with the medium and high groups. These higher levels of HSP20 and HSP27 are explained by studies conducted by Morzel et al. [32] and Lomiwes et al. [31], who reported that myofibrillar protein aggregation inhibits protein degradation caused by endogenous proteases, and higher levels of HSP20 and HSP27 prevent the formation of aggregated protein complexes. In addition, the chaperone property of HSP20 and HSP27 in the low group may be associated with lower cooking loss compared to the high group. However, the levels of HSP70 and HSP90 at the early postmortem period had a weak association with the WBSF value and palatability of highly marbled Hanwoo steers, although the function of these proteins is to protect muscle fibers from various stressors, including oxidative stress and chemotherapeutic agents [33]. Therefore, the lower level of αβ-crystallin and higher levels of HSP20 and HSP27 at the early postmortem period are associated with lower WBSF value and higher scores of sensory tenderness attributes in highly marbled muscle samples from the low group.

HSPs appear in myofibril within 30 min of stress being initiated [2,9]. During the postmortem period, as muscle pH declines to the isoelectric points of individual HSPs, the disappearance of HSPs due to protein precipitation is accelerated, and protein precipitates are transferred from the sarcoplasmic to myofibrillar fraction [9]. The concentrations of these proteins in the sarcoplasmic fraction reach minimum values within 48 h postmortem [2,9]. Lomiwes et al. [5] suggested that lower shear force values were associated with the precipitation and degradation of small HSPs. In the current study, the disappearance of αβ-crystallin and HSP90 from the sarcoplasmic fraction due to protein degradation was observed at 24 h postmortem, although the concentrations of HSPs at 24 h postmortem were not associated with the WBSF value and sensory quality characteristics of highly marbled Hanwoo steer. This result can be explained by a previous study [31], which reported that tenderness of cooked beef was not affected by the amount of HSPs at 24 h postmortem due to impaired biological functions of these proteins.

## 5. Conclusions

Taken together, lower level of αβ-crystallin and higher levels of HSP20 and HSP27 at 45 min postmortem were associated with a greater tenderness of cooked beef measured by instrumental and sensory quality evaluations. Therefore, the levels of small HSPs at the early postmortem period can be relevant indicators to explain the tenderness variation in highly marbled LT muscle from Hanwoo steers, which exhibit low pH_24 h_.

## Figures and Tables

**Table 1:** Comparison of Warner–Bratzler shear force (WBSF), marbling score, intramuscular fat (IMF) content, and carcass characteristics of the Hanwoo *longissimus thoracis* muscle in groups categorized by WBSF value.

	WBSF Value Group			Level of Significance ^1^
	Low (*n* = 17)	Medium (*n* = 18)	High (*n* = 14)	
WBSF (N)	37.8 ^c^ (1.04) ^2^	48.9 ^b^ (0.92)	64.3 ^a^ (1.29)	***
Marbling score	7.76 (0.15)	7.34 (0.15)	7.29 (0.17)	NS
IMF content (%)	20.9 (1.35)	19.2 (1.01)	19.6 (0.96)	NS
Carcass characteristics				
Carcass weight (kg)	470 (15.8)	480 (15.4)	445 (17.4)	NS
Loin eye area (cm^2^)	103 (3.44)	101 (3.35)	101 (3.79)	NS
Back-fat thickness (mm)	18.8 ^a^ (1.53)	19.5 ^a^ (1.48)	13.8 ^b^ (1.68)	*

^1^ Level of significance: NS = not significant; * *p* < 0.05; *** *p* < 0.001. ^a–c^ Different superscript letters in the same row represent significant differences (*p* < 0.05). ^2^ Standard error of least-square means.

**Table 2:** Comparison of meat quality characteristics of the Hanwoo *longissimus thoracis* muscle in groups categorized by Warner–Bratzler shear force (WBSF) value.

	WBSF Value Group			Level of Significance ^1^
	Low (*n* = 17)	Medium (*n* = 18)	High (*n* = 14)	
Muscle pH				
Muscle pH_45__min_	6.52 (0.08) ^2^	6.44 (0.06)	6.46 (0.06)	NS
Muscle pH_24__h_	5.51 (0.05)	5.46 (0.04)	5.48 (0.03)	NS
Meat color				
Lightness (*L* ^*^)	31.7 (1.76)	33.4 (1.19)	31.2 (1.09)	NS
Redness (*a* ^*^)	17.0 (1.60)	16.5 (1.08)	16.6 (0.99)	NS
Yellowness (*b* ^*^)	7.44 (1.06)	7.66 (0.71)	7.12 (0.66)	NS
Water-holding capacity				
Drip loss (%)	1.25 (0.38)	0.93 (0.28)	1.45 (0.26)	NS
Filter-paper fluid uptake (mg)	4.35 (1.28)	2.81 (0.94)	4.65 (0.87)	NS
Cooking loss (%)	17.1 ^b^ (1.15)	19.9 ^ab^ (1.05)	21.7 ^a^ (1.48)	*

^1^ Level of significance: NS = not significant; * *p* < 0.05. ^a–b^ Different superscript letters in the same row represent significant differences (*p* < 0.05). ^2^ Standard error of least-square means.

**Table 3:** Comparison of sensory quality characteristics of cooked beef in groups categorized by Warner–Bratzler shear force (WBSF) value.

	WBSF Value Group			Level of Significance ^1^
	Low (*n* = 17)	Medium (*n* = 18)	High (*n* = 14)	
Tenderness attributes ^3^				
Softness	7.49 ^a^ (0.19) ^2^	6.89 ^b^ (0.18)	6.16 ^c^ (0.22)	***
Initial tenderness	7.11 ^a^ (0.20)	6.75 ^a^ (0.19)	6.05 ^b^ (0.24)	***
Chewiness	6.78 ^a^ (0.22)	6.40 ^a^ (0.22)	5.85 ^b^ (0.27)	*
Rate of breakdown	6.67 ^a^ (0.19)	6.19 ^a^ (0.19)	5.35 ^b^ (0.23)	***
Amount of perceptible residue	6.88 ^a^ (0.18)	6.53 ^a^ (0.17)	5.94 ^b^ (0.21)	**
Mouth coating	6.97 ^a^ (0.22)	6.34 ^b^ (0.21)	6.26 ^b^ (0.26)	**
Juiciness	6.69 ^a^ (0.19)	6.40 ^a^ (0.19)	5.85 ^b^ (0.23)	**
Flavor intensity	6.49 (0.14)	6.31 (0.14)	6.06 (0.17)	NS
Off-flavor intensity	6.92 (0.12)	6.67 (0.12)	6.63 (0.14)	NS

^1^ Level of significance: NS = not significant; * *p* < 0.05; ** *p* < 0.01; *** *p* < 0.001. ^a–c^ Different superscript letters in the same row represent significant differences (*p* < 0.05). ^2^ Standard error of least-square means. ^3^ Score distribution: low to high, softness: hard to soft; initial tenderness: tough to tender; chewiness: very chewy to very tender; rate of breakdown: very slow to very fast; amount of perceptible residue: abundant to none; mouth coating: none to very high; juiciness: not juicy to extremely juicy; flavor intensity: very weak to very strong; off-flavor intensity: very strong to very weak.
